# Incidence and risk factors of acute kidney injury among neurocritical patients in high-altitude regions: a retrospective cohort study

**DOI:** 10.3389/fmed.2026.1756455

**Published:** 2026-04-22

**Authors:** Quzhen Danzeng, Yingyu Pan, Zihan Lin, Jinwei Wang, Nan Li, Guoying Lin, Dong Wu

**Affiliations:** 1 Department of Intensive Care Unit, People’s Hospital of Xizang Autonomous Region, Lhasa, China; 2 Department of Gastroenterology, Peking Union Medical College Hospital, Chinese Academy of Medical Sciences and Peking Union Medical College, Beijing, China; 3 Renal Division, Department of Medicine, Peking University First Hospital, Beijing, China; 4 Department of Intensive Care Unit, Peking University First Hospital, Beijing, China; 5 Department of Gastroenterology, People’s Hospital of Xizang Autonomous Region, Lhasa, China

**Keywords:** acute kidney injury, brain injury, critical care, erythrocytosis, high-altitude

## Abstract

**Background:**

Acute kidney injury (AKI) is a frequent complication in intensive care unit (ICU) patients with acute brain injury. High-altitude hypoxia may aggravate renal vulnerability, but relevant evidence is limited. This study aims to investigate the incidence, risk factors, and outcomes of AKI among neurocritical patients at high altitude.

**Methods:**

Adult neurocritical patients admitted to the ICU of People’s Hospital of Xizang Autonomous Region (3,650 m) from January 2022 to February 2024 were retrospectively analyzed. AKI was defined using the 2012 KDIGO serum creatinine criteria. Multivariable logistic regression identified independent predictors, and hemoglobin levels were modeled with linear spline regression. Propensity score matching (PSM) was performed to evaluate the impact of specific interventions.

**Results:**

Among 390 patients, 83 (21.3%) developed AKI within 7 days of ICU admission. Independent predictors included older age (OR = 1.058 [1.032–1.087], *p* < 0.001), higher body mass index (OR = 1.130 [1.030–1.242], *p* = 0.010), lower Glasgow Coma Scale score (OR = 0.859 [0.795–0.924], *p* < 0.001), hypotension (OR = 2.659 [1.257–5.579], *p* = 0.010), contrast agent (OR = 2.480 [1.263–4.867], *p* = 0.008), high-dose loop diuretics (OR = 1.985 [1.117–3.555], *p* = 0.020), and baseline eGFR (OR = 1.033 [1.015–1.052], *p* < 0.001). Both low (<100 g/L, OR = 4.707 [1.020–19.686], *p* = 0.037) and high (>180 g/L, OR = 2.031 [1.093–3.790], *p* = 0.025) hemoglobin levels increased AKI risk. PSM sensitivity analysis confirmed the association of high-dose loop diuretics with AKI (OR = 2.009, *p* = 0.011), while the effect of contrast agents lost significance after matching (OR = 1.505, *p* = 0.215). Patients with AKI had higher in-hospital mortality (34.9% vs. 11.7%, *p* < 0.001) and greater hospitalization costs (148.5 vs. 119.4 × 1,000 CNY, *p* = 0.001).

**Conclusion:**

At high altitude, AKI is common in neurocritical patients and independently associated with adverse outcomes. Beyond traditional risk factors, altitude-related erythrocytosis significantly contribute to AKI risk, suggesting that tailored renal protection strategies are necessary in high-altitude neurocritical care.

## 1 Introduction

Acute kidney injury (AKI) is one of the most common organ dysfunctions in intensive care unit (ICU) patients. It is an independent risk factor for mortality in ICU and is associated with increased morbidity and medical costs (1). Patients with acute brain injury (ABI) are at particularly high risk of developing AKI, with previous studies reporting an incidence exceeding 20% among neurocritical care patients (2). In addition to common risk factors such as sepsis, hypovolemia, chronic cardiovascular disease and advanced age (3), neurological critical illness introduces specific pathological mechanisms and therapeutic interventions that may further contribute to AKI development. ABI compromises the integrity of the blood–brain barrier, triggering the systemic release of pro-inflammatory cytokines that induce renal tubular epithelial cell injury or dysfunction (4, 5). Furthermore, the intense activation of the sympathetic nervous system post-ABI leads to pronounced renal vasoconstriction and stimulates the renin-angiotensin-aldosterone system, culminating in functional renal impairment (6). Routine clinical management in neurocritical care often serves as an additional trigger for renal insult. Hyperosmolar therapies (e.g., mannitol or hypertonic saline) used to control intracranial pressure can induce osmotic nephrosis, while high-dose vasopressors required to maintain cerebral perfusion pressure may inadvertently sacrifice renal perfusion (7–9). The occurrence and severity of AKI in neurocritical patients are closely associated with increased mortality, impaired neurological recovery, greater disability at discharge, and higher long-term healthcare burden (1).

**Figure 1:**
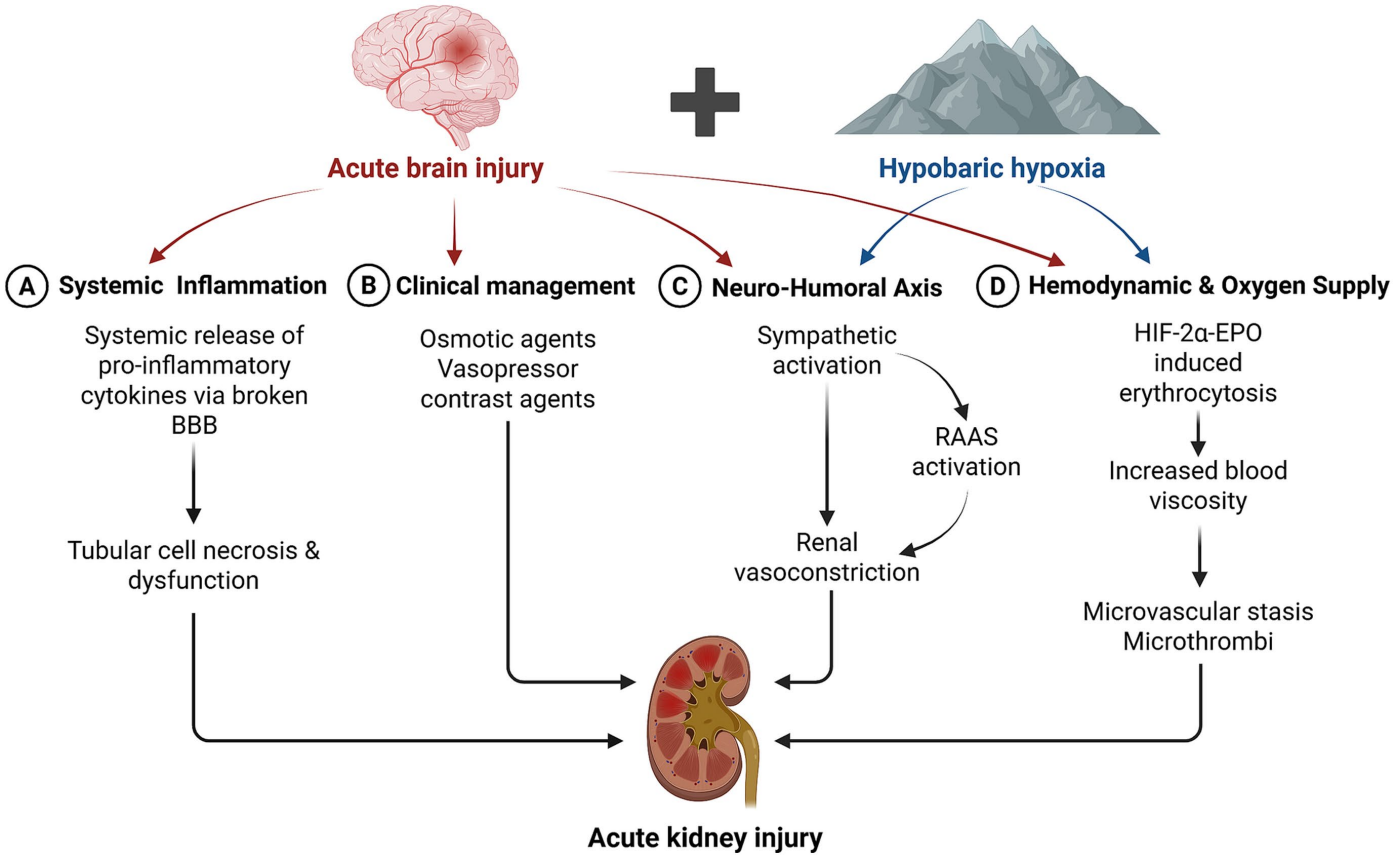
Pathophysiological framework of acute kidney injury in high-altitude neurocritical patients. BBB, blood–brain barrier; RAAS, renin-angiotensin-aldosterone system; HIF-2α, hypoxia-inducible factor 2α; EPO, erythropoietin.

High altitude is generally defined as areas with an elevation of over 1,500 meters above sea level. For neurocritical patients in these regions, AKI does not simply result from two independent pathological conditions but rather from the complex and mutually reinforcing interaction between systemic alterations caused by neurological injury and the physiological adaptations to hypobaric hypoxia. On the one hand, compared with lowland regions, patients with ABI at high altitude tend to have more severe disease and a higher propensity for multi-organ injury involving the heart, liver, and kidneys (10, 11). On the other hand, the hypoxic environment at high altitude further amplifies sympathetic excitation, exacerbating renal vasoconstriction. Moreover, hypoxia-inducible factor (HIF) signaling pathway is activated under hypoxic conditions. HIF-2α serves as the master regulator of chronic adaptive responses, which upregulates the transcription of erythropoietin (EPO), stimulating red blood cell production and raising hemoglobin levels (12). However, when this response is excessive, it leads to high-altitude polycythemia (HAPC), clinically defined by the Qinghai Criteria as a hemoglobin concentration of >210 g/L in males and >190 g/L in females. While increased hemoglobin initially compensate for hypoxia, it cause a exponential rise in whole-blood viscosity, which significantly impairs renal microcirculatory flow, predisposing patients to microthrombi formation and microcirculatory stasis, and further impairing renal oxygen delivery (13, 14) (Figure 1).

To date, evidence regarding AKI among neurocritical patients in high-altitude regions remains limited, hindering a comprehensive understanding of this distinct clinical condition. People’s Hospital of Xizang Autonomous Region, located on the world’s highest plateau, is the largest and most representative medical center in the region. This study aimed to systematically investigate the incidence, risk factors, and outcomes of AKI among neurocritical patients admitted to its ICU, providing valuable clinical evidence to improve renal outcomes in high-altitude settings.

## 2 Materials and methods

### 2.1 Sample source

**Figure 2:**
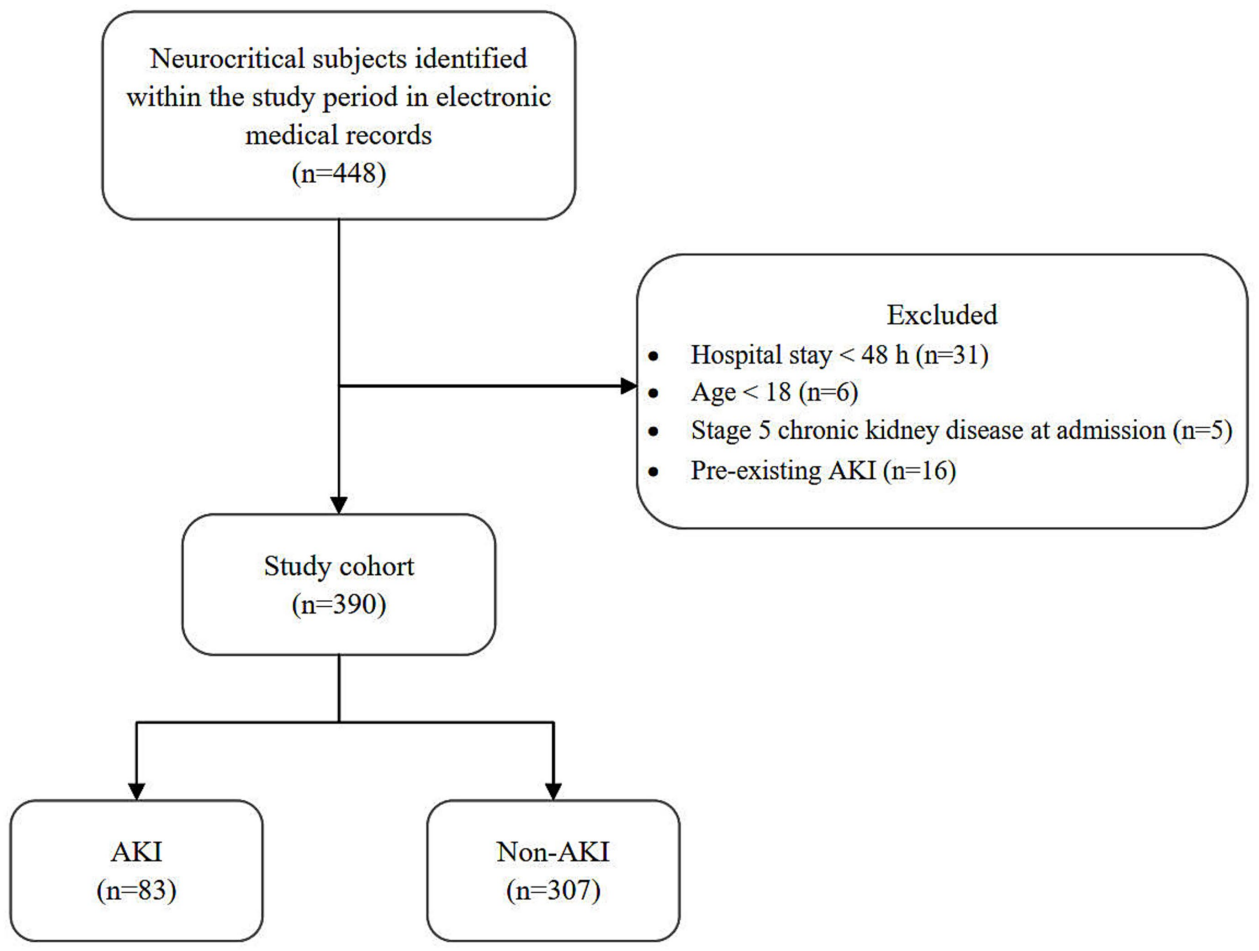
Flow diagram of patients enrolled in this study.

This retrospective study included adult neurocritical patients admitted to ICU of People’s Hospital of Xizang Autonomous Region between January 2022 and February 2024. Neurocritical conditions were categorized into three groups: (1) traumatic brain injury; (2) cerebrovascular disorders, including hemorrhagic stroke, ischemic stroke, hypertensive intracerebral hemorrhage, and aneurysmal subarachnoid hemorrhage; and (3) other neurological diseases such as intracranial mass lesions, central nervous system infections, status epilepticus, and post-cardiac arrest syndromes. Patients were excluded if they had a hospital stay shorter than 48 h, pre-existing AKI before ICU admission, stage 5 chronic kidney disease, or end-stage renal disease requiring maintenance dialysis. During the study period, 448 neurocritical patients were admitted to the ICU, and 390 met the inclusion criteria (Figure 2). The study was approved by Ethics Committee of People’s Hospital of Xizang Autonomous Region (No. ME-TBHP-24-057).

### 2.2 Data collection

The primary endpoint was the occurrence of AKI within 7 days after ICU admission. AKI was defined according to 2012 Kidney Disease: Improving Global Outcomes (KDIGO) criteria. The baseline serum creatinine (SCr) was determined using a hierarchical approach: (1) the lowest SCr value within 3 months prior to admission; (2) if unavailable, the first SCr value measured upon hospital admission; (3) for patients with no prior SCr records and no known history of kidney disease, the baseline SCr was back-calculated using the Modification of Diet in Renal Disease (MDRD) formula, assuming a baseline eGFR of 75 mL/min/1.73m^2^. The severity of AKI was classified based on the 2012 KDIGO SCr staging criteria. Secondary endpoints included duration of mechanical ventilation, ICU length of stay, total hospital length of stay, in-hospital mortality, and total hospitalization cost. Relevant clinical data of all enrolled patients were collected from the hospital electronic medical record system. To better characterize the role of nephrotoxic exposures as potential confounders, the cumulative doses of these agents within 72 h of ICU admission were also extracted, as the majority of AKI episodes occurred within this timeframe. Due to clinical record limitations, specific doses of contrast agents could not be accurately obtained, and hence contrast exposure was analyzed as a categorical variable.

### 2.3 Statistical analysis

Continuous variables were expressed as mean ± standard deviation (mean ± SD) for normally distributed data or as median (interquartile range, IQR) for non-normally distributed data. Normality was assessed using the Shapiro–Wilk test. Between-group comparisons were conducted using the *t*-test for normally distributed variables and the Mann–Whitney *U* or Kruskal–Wallis test for non-normally distributed variables. Categorical variables were presented as frequencies and percentages, and compared using the chi-square test or Fisher’s exact test when expected counts were <5. No missing value was included in the data analysis.

To explore the potential non-linear relationship between hemoglobin levels and AKI, linear spline regression was performed with knots at the 25th, 50th, and 75th percentiles. The reference value for the odds ratio (OR) was set at 120 g/L, which aligns with a clinically normal physiological baseline. Uncertainty estimates for the spline model were calculated and presented as 95% confidence intervals (CIs). Potential risk factors for AKI were first screened using univariate logistic regression. Variables with *p* < 0.05 or clinical relevance were entered into a multivariate model via backward likelihood ratio selection. Multicollinearity was considered absent if the variance inflation factor (VIF) was <5. Model discrimination was evaluated by the area under the curve (AUC), and calibration was assessed using the Hosmer–Lemeshow goodness-of-fit test. A propensity score matching (PSM) sensitivity analysis was performed to mitigate confounding by indication regarding specific intensive care interventions, namely contrast agent exposure and the use of high-dose loop diuretics. High-dose loop diuretics were defined as a cumulative furosemide dose > 25 mg within 72 h of ICU admission, representing the median dose among users. For contrast exposure, patients were matched 1:2 using a nearest-neighbor algorithm without replacement (caliper = 0.2) based on *a priori* selected covariates (age, sex, BMI, hypertension, cerebrovascular injury, GCS, APACHE II, baseline eGFR, and hemoglobin categories). Similarly, 1:1 matching was applied for high-dose loop diuretic exposure using relevant baseline covariates (age, sex, BMI, hypertension, cerebrovascular injury, GCS, APACHE II, baseline eGFR, hemoglobin categories, and hypotension). Covariate balance was confirmed if the standardized mean difference (SMD) was <0.1, and the AKI risk was subsequently re-evaluated in the matched cohorts. Kaplan–Meier curves were generated to illustrate in-hospital mortality within 30 days after ICU admission according to AKI severity, and survival differences between groups were compared using the log-rank test. Patients who were discharged or remained alive after 30 days were considered censored.

All statistical analyses were performed using Stata Statistical Software version 16 (StataCorp, TX, United States) and R version 4.4.1 (R Foundation for Statistical Computing, Vienna, Austria).

## 3 Results

A total of 390 patients were included in the study, and 243 (62.3%) were male. Most patients had normal renal function during hospitalization, while 83 (21.3%) developed AKI within 7 days after ICU admission. Among AKI cases, stage 1 accounted for 50.6%, stage 2 for 25.3%, and stage 3 for 24.1%. In 62 (74.7%) of these patients, AKI occurred within 72 h of ICU admission.

Baseline characteristics of patients with and without AKI were summarized in Table 1. Compared with non-AKI patients, those with AKI were older (55 ± 13 vs. 50 ± 15 years, *p* = 0.004), and had a higher proportion of males (73.5% vs. 59.3%, *p* = 0.018), higher body mass index (BMI) (24.6 ± 3.2 vs. 23.8 ± 3.2 kg/m^2^, *p* = 0.038), lower Glasgow Coma Scale (GCS) scores on admission (6 [5, 9] vs. 11 [6, 15], *p* < 0.001). Cerebrovascular injury was the most common type of craniocerebral injury in both groups but was more frequent among AKI patients (81.9% vs. 64.2%, *p* < 0.05). Correspondingly, hypertension was the most prevalent comorbidity overall and was more common in the AKI group (61.4% vs. 38.4%, *p* < 0.001).

**Table 1:** Baseline characteristics of neurocritical patients with and without acute kidney injury.

Variables	All (*n* = 390)	Non-AKI (*n* = 307)	AKI (*n* = 83)	*p*
Male	243 (62.3%)	182 (59.3%)	61 (73.5%)	0.018*
Age (y)	51 ± 15	50 ± 15	55 ± 13	0.004*
BMI (kg/m^2^)	24.0 ± 3.2	23.8 ± 3.2	24.6 ± 3.2	0.038*
Tibetan	300 (76.9%)	237 (77.2%)	63 (75.9%)	0.804
Types of craniocerebral injury				0.008*
Traumatic brain injury	52 (13.3%)	45 (14.7%)	7 (8.4%)	
Cerebrovascular injury^a^	265 (67.9%)	197 (64.2%)	68 (81.9%)	
Other^b^	73 (18.7%)	65 (21.2%)	8 (9.6%)	
GCS score on admission	9 (5,15)	11 (6,15)	6 (5,9)	<0.001*
Comorbidity				
Hypertension	169 (43.3%)	118 (38.4%)	51 (61.4%)	<0.001*
Diabetes mellitus	6 (1.5%)	4 (1.3%)	2 (2.4%)	0.612
Coronary artery disease	2 (0.5%)	1 (0.3%)	1 (1.2%)	0.381
Chronic kidney disease	0 (0.0%)	0 (0.0%)	0 (0.0%)	–
Lab tests on admission				
Albumin (g/L)	40.0 ± 6.0	39.8 ± 5.9	40.7 ± 6.4	0.231
Hemoglobin (g/L)	161 (145, 185)	160 (144, 180)	173 (154, 201)	0.001*
<100	14 (3.6%)	10 (3.3%)	4 (4.8%)	0.002*
100–180	267 (68.5%)	223 (72.6%)	44 (53.0%)	
>180	109 (27.9%)	74 (24.1%)	35 (42.2%)	
MCHC (g/L)	336 ± 16	335 ± 16	338 ± 16	0.189
HCT (%)	47.9 (43.5, 55.3)	47.5 (42.8, 53.2)	51.2 (45.6, 58.8)	<0.001*
>65	35 (9.0%)	25 (8.1%)	10 (12.0%)	0.375
Na^+^ (mmol/L)	139 (136, 141)	139 (136, 141)	139 (136, 141)	0.283
BUN/creatinine ratio	18.0 (14.4, 23.0)	17.7 (14.3, 22.7)	19.3 (15.4, 24.7)	0.052
>20	140 (35.9%)	104 (33.9%)	36 (43.4%)	0.141
Baseline serum creatinine (μmol/L)	60.5 ± 17.9	61.2 ± 16.6	58.1 ± 21.9	0.245
Baseline eGFR (mL/min·1.73m^2^)	106 (94, 120)	105 (92, 122)	106 (94, 119)	0.976
Operation	335 (85.9%)	264 (86.0%)	71 (85.5%)	0.917

AKI, acute kidney injury; BMI, body mass index; GCS, Glasgow Coma Scale; MCHC, mean corpuscular hemoglobin concentration; BUN, blood urea nitrogen; eGFR, estimated glomerular filtration rate.

^a^Including hemorrhagic stroke, ischemic stroke, hypertensive intracerebral hemorrhage, and aneurysmal subarachnoid hemorrhage.

^b^Including intracranial space-occupying lesions, intracranial infections, status epilepticus, and post-cardiopulmonary resuscitation.

*Statistical significant.

**Figure 3:**
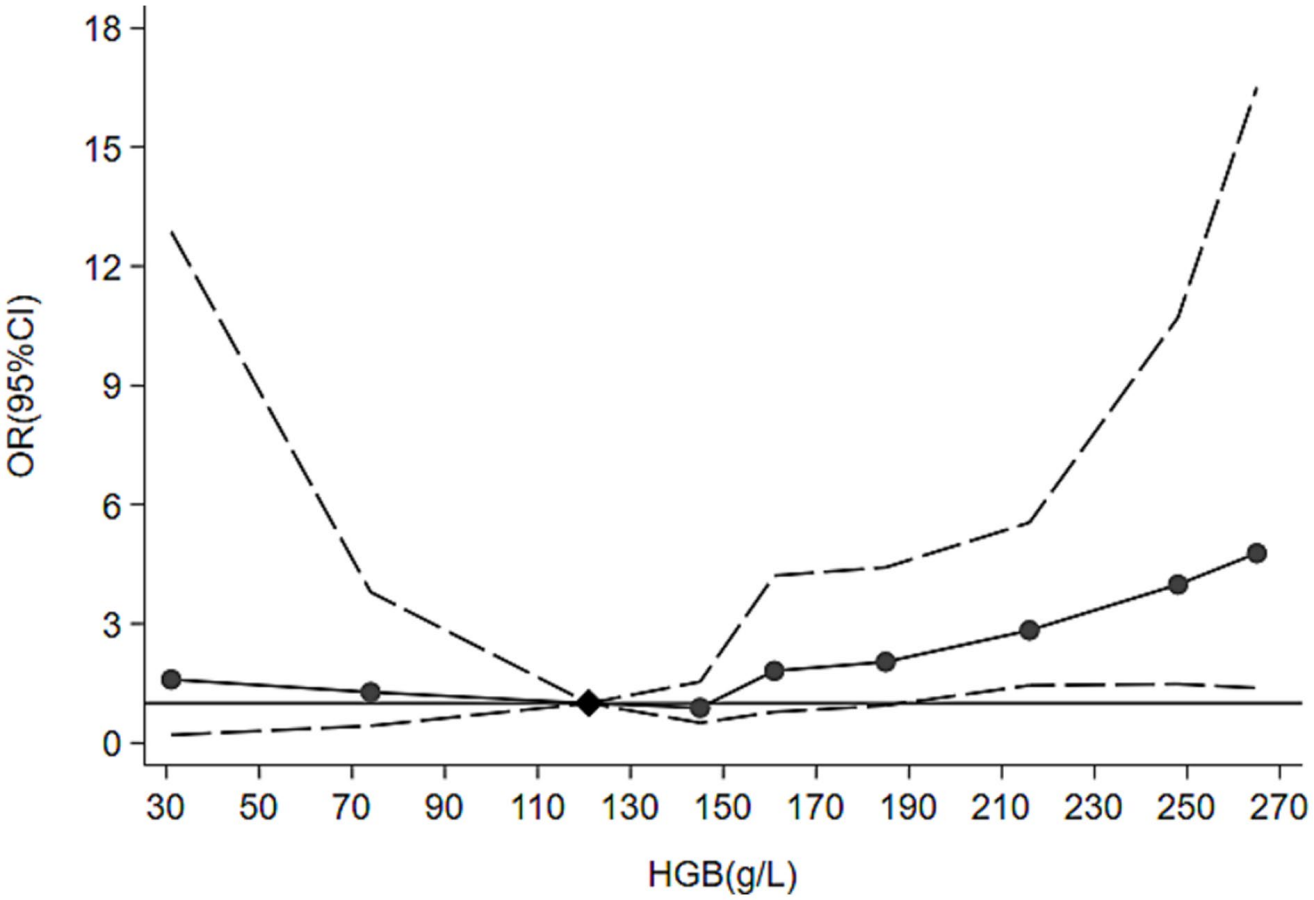
Association between hemoglobin levels and the risk of acute kidney injury. HGB, hemoglobin; OR, odds ratio; CI, confidence interval. Hemoglobin was modeled as a linear spline with knots at the 25th, 50th, and 75th percentiles, using logistic regression. The odds ratio was estimated with 120 g/L as the reference point (odds ratio = 1), and the dashed lines indicated the 95% confidence interval. Both low and high hemoglobin levels were associated with an increased risk of acute kidney injury, and the risk rose progressively as hemoglobin deviated from the reference value.

Notably, hemoglobin levels were significantly higher in the AKI group than in the non-AKI group (173 [154, 201] vs. 160 [144, 180], *p* = 0.001). To further explore the relationship between hemoglobin and AKI risk, spline regression analysis was performed using 120 g/L as the reference point (Figure 3). Both low and high hemoglobin levels were associated with increased AKI risk, and the strength of this association rose progressively as hemoglobin deviated from the reference range. Based on the inflection points observed in the spline model, where the point estimates of risk sharply increased and the 95% CIs progressively deviated from the reference line, combined with established clinical thresholds for moderate anemia and erythrocytosis, hemoglobin was categorized into three distinct groups (<100, 100–180, and >180 g/L) for subsequent analysis. Within this framework, elevated hemoglobin (>180 g/L) was observed in 42.2% of AKI patients, compared with 24.1% of non-AKI patients (*p* < 0.05) (Table 1).

Differences in clinical status at ICU admission were shown in Table 2. Patients with AKI had significantly higher APACHE II scores (18.3 ± 5.3 vs. 15.6 ± 5.5, *p* < 0.001), and a higher incidence of hypotension (22.9% vs. 12.1%, *p* = 0.012) and vasopressor use (71.1% vs. 58.6%, *p* = 0.039). Nephrotoxic agents such as mannitol (93.6%), loop diuretics (86.4%), nonsteroidal anti-inflammatory drugs (20.0%), vancomycin (11.3%), and contrast agents (21.8%) were administered or continued during ICU stay. Only the use of contrast agents showed a significant difference between groups (30.1% vs. 19.5%, *p* = 0.038). Additionally, while the overall usage rates of mannitol, loop diuretics, NSAIDs, and vancomycin were comparable between groups, the cumulative dose of furosemide was significantly higher in the AKI group (40 [20, 70] vs. 20 [10, 40] mg, *p* < 0.001) (Supplementary Table S1).

**Table 2:** Clinical features and management at intensive care unit in neurocritical patients with and without acute kidney injury.

Variables	All (*n* = 390)	Non-AKI (*n* = 307)	AKI (*n* = 83)	*p*
APACHE II score	16.2 ± 5.6	15.6 ± 5.5	18.3 ± 5.3	<0.001*
Hypotension^a^	56 (14.4%)	37 (12.1%)	19 (22.9%)	0.012*
Vasopressor infusion^b^	239 (61.3%)	180 (58.6%)	59 (71.1%)	0.039*
Mechanical ventilation	390 (100%)	307 (100%)	83 (100%)	-
Nephrotoxic agents				
Mannitol	365 (93.6%)	287 (93.5%)	78 (94.0%)	0.871
Loop diuretics	337 (86.4%)	260 (84.7%)	77 (92.8%)	0.057
NSAIDs	78 (20.0%)	59 (19.2%)	19 (22.9%)	0.458
Vancomycin	44 (11.3%)	31 (10.1%)	13 (15.7%)	0.155
Contrast agents	85 (21.8%)	60 (19.5%)	25 (30.1%)	0.038*

AKI, acute kidney injury; APACHE II, Acute Physiology and Chronic Health Evaluation II; NSAIDs, non-steroidal anti-inflammatory drugs.

^a^Defined as a mean arterial pressure < 65 mmHg.

^b^Including norepinephrine, dopamine, and metaraminol.

*Statistical significant.

Univariate analyses of potential risk factors for AKI were conducted (Table 3). Multivariate logistic regression using the backward likelihood ratio method identified age (OR = 1.058 [1.032–1.087], *p* < 0.001), BMI (OR = 1.130 [1.030–1.242], *p* = 0.010), GCS score on admission (OR = 0.859 [0.795–0.924], p < 0.001), hypotension (OR = 2.659 [1.257–5.579], *p* = 0.010), use of contrast agents (OR = 2.480 [1.263–4.867], *p* = 0.008), high-dose loop diuretics (OR = 1.985 [1.117–3.555], *p* = 0.020), and baseline eGFR (OR = 1.033 [1.015–1.052], *p* < 0.001) as independent risk factors of AKI in neurocritical patients. Particularly, both lowered (<100 g/L, OR = 4.707 [1.020–19.686], *p* = 0.037) and elevated (>180 g/L, OR = 2.031 [1.093–3.790], *p* = 0.025) hemoglobin were associated with increased risk of AKI. The final multivariate model demonstrated good discrimination (AUC = 0.822) and adequate calibration (Hosmer–Lemeshow test *p* = 0.118). Multicollinearity was absent.

**Table 3:** Univariate and multivariate Logistic regression analyses of risk factors for acute kidney injury.

Variables	Univariate		Multivariate	
	OR (95%CI)	*p*	OR (95%CI)	*p*
Age (y)	1.025 (1.008–1.044)	0.005	1.058 (1.032–1.087)	<0.001
Male	1.904 (1.126–3.318)	0.019	1.939 (0.911–3.838)	0.061
BMI (kg/m^2^)	1.079 (1.003–1.161)	0.041	1.130 (1.030–1.242)	0.010
Cerebrovascular injury^a^	2.531 (1.415–4.790)	0.003	–	–
GCS score on admission	0.861 (0.810–0.912)	<0.001	0.859 (0.795–0.924)	<0.001
Hypertension	2.553 (1.559–4.234)	<0.001	–	–
Hemoglobin (g/L)				
<100	2.027 (0.537–6.367)	0.250	4.707 (1.020–19.686)	0.037
100–180	Reference	–	Reference	–
>180	2.397 (1.427–4.016)	0.001	2.031 (1.093–3.790)	0.025
APACHE II score	1.089 (1.042–1.140)	<0.001	1.041 (0.986–1.100)	0.147
Hypotension^b^	2.166 (1.152–3.978)	0.014	2.659 (1.257–5.579)	0.010
Use of contrast agents	1.774 (1.016–3.046)	0.040	2.480 (1.263–4.867)	0.008
Vasopressor infusion^c^	1.734 (1.036–2.979)	0.040	–	–
High-dose loop diuretics^d^	2.991 (1.818–4.998)	<0.001	1.985 (1.117–3.555)	0.020
Baseline eGFR (mL/min·1.73m^2^)	1.007 (0.995–1.020)	0.247	1.033 (1.015–1.052)	<0.001

OR, odds ratio; CI, confidence interval; BMI, body mass index; GCS, Glasgow Coma Scale; APACHE II, Acute Physiology and Chronic Health Evaluation II.

^a^Including hemorrhagic stroke, ischemic stroke, hypertensive intracerebral hemorrhage, and aneurysmal subarachnoid hemorrhage.

^b^Defined as a mean arterial pressure < 65 mmHg.

^c^Including norepinephrine, dopamine, and metaraminol.

^d^Furosemide > 25 mg within 72 h of ICU admission.

In the PSM cohort for contrast exposure (Supplementary Table S2), the association between contrast agent administration and AKI risk was attenuated and no longer statistically significant (OR = 1.505 [0.788–2.875], *p* = 0.215). Conversely, after PSM for high-dose loop diuretics (Supplementary Table S3), the association remained significant, with high-dose furosemide independently associated with increased risk of AKI (OR = 2.009 [1.177–3.429], *p* = 0.011).

**Figure 4:**
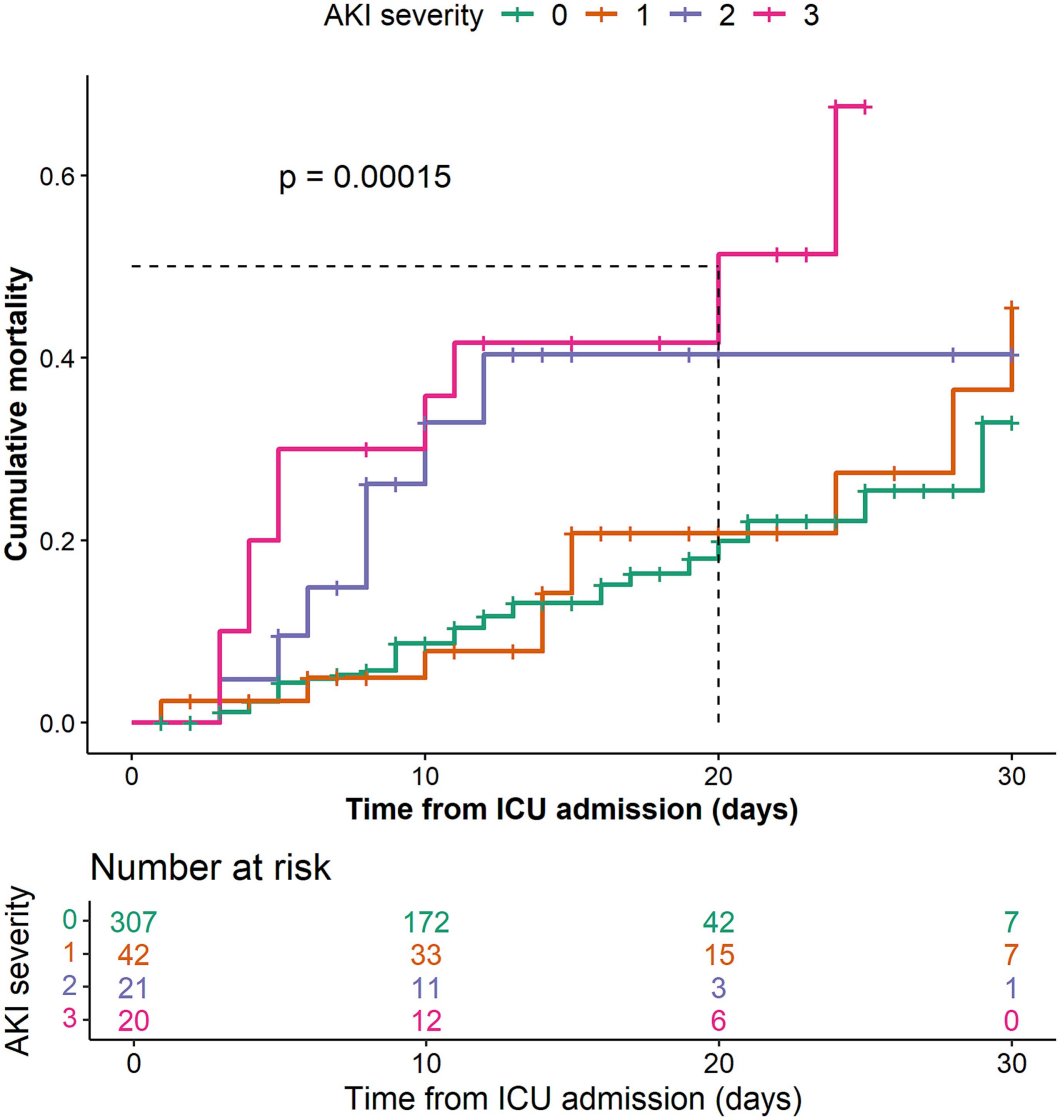
Kaplan–Meier survival curves by acute kidney injury severity showing time from intensive care unit admission to death or censoring (30 days or discharge).

Regarding clinical outcomes, all patients required mechanical ventilation during ICU stay. The duration of ventilation was significantly longer in the AKI group (266 [122, 347] vs. 177 [72, 280] hours, *p* < 0.001). AKI patients also had longer ICU stays (14 [8, 22] vs. 11 [5, 16] days, *p* = 0.002), higher in-hospital mortality (34.9% vs. 11.7%, *p* < 0.001), and greater hospitalization costs (148.5 [88.8, 234.1] vs. 119.4 [70.3, 169.1] × 1,000 CNY, *p* = 0.001) compared with those without AKI (Table 4). The Kaplan–Meier curves showed a significant difference in mortality risk among patients with different AKI stages (*p* = 0.00015), with higher AKI stages associated with poorer outcomes (Figure 4).

**Table 4:** Outcomes of neurocritical patients with and without acute kidney injury.

Variables	All (*n* = 390)	Non-AKI (*n* = 307)	AKI (*n* = 83)	*p*
Duration of mechanical ventilation (h)	194 (89, 300)	177 (72, 280)	266 (122, 347)	<0.001*
ICU length of stay (d)	11 (6, 17)	11 (5, 16)	14 (8, 22)	0.002*
Total hospital length of stay (d)	27 (15, 45)	26 (16,41)	28 (11, 68)	0.402
In-hospital mortality	65 (16.7%)	36 (11.7%)	29 (34.9%)	<0.001*
Total hospitalization cost (1,000 yuan)	125.8 (74.5, 185.0)	119.4 (70.3, 169.1)	148.5 (88.8, 234.1)	0.001*

AKI, acute kidney injury; ICU, intensive care unit.

*Statistical significant.

AKI, acute kidney injury; ICU intensive care unit.

## 4 Discussion

The AKI in neurocritical patients represents a major clinical challenge, especially in high-altitude regions where environmental hypoxia may interact with neurological and systemic pathophysiological changes. Understanding epidemiology and risk factors of AKI in this unique population is crucial for improving early recognition and renal protection strategies. To our knowledge, this is the first study focusing on AKI among neurocritical patients living at high altitude.

In this study conducted at an elevation of 3,650 m in Lhasa, Xizang, the incidence of AKI among neurocritical care patients was high (21.3%). It should be noted that urine output data were not available from the electronic medical records, and thus AKI was defined solely based on SCr, which may have led to an underestimation of its true incidence. Evidence regarding the prevalence of AKI in neurocritical care is limited. A retrospective study from Chile reported an overall AKI incidence of 23.5% when applying KDIGO criteria based on either SCr or urine output, which is comparable to our findings (2). Moreover, patients with AKI in our study had worse outcomes, including longer durations of mechanical ventilation and ICU stay, higher in-hospital mortality, and increased hospitalization costs. These results highlight the importance of early prevention and close monitoring of AKI in neurocritical care to improve clinical outcomes.

Our findings demonstrated that both elevated (>180 g/L) and reduced (<100 g/L) hemoglobin levels were associated with a higher risk of AKI. The relationship between hemoglobin levels and AKI has been widely reported in both neurocritical (15, 16) and other clinical settings (17–19). Anemia is thought to increase AKI susceptibility because the kidney, a highly perfused organ, is extremely sensitive to hypoxia. Reduced oxygen delivery caused by low hemoglobin may predispose renal tissue to ischemic injury when other stressors are present.

As the first study to examine this issue at high altitude, we found that elevated hemoglobin levels were significantly associated with an increased risk of AKI. While the comparable mean corpuscular hemoglobin concentration (MCHC) between groups helps exclude hemolysis-induced nephrotoxicity, the erythrocytosis hypothesis is further supported by our hematocrit (HCT) analysis. Specifically, patients in the AKI group demonstrated significantly higher HCT levels compared to the non-AKI group (51.2 [45.6, 58.8] vs. 47.5 [42.8, 53.2], *p* < 0.001). Chronic hypoxia at high altitude induces compensatory erythrocytosis, a core driver of HAPC and the subsequent High-Altitude Renal Syndrome (HARS) (14). Although HARS typically represents a chronic process leading to progressive renal injury (20), our findings suggest that erythrocytosis may also exert acute effects, predisposing the kidney to injury under additional systemic stress.

The operative mechanism likely involves increased blood viscosity, which can impair renal hemodynamics (21). Experimental data have confirmed that acute polycythemia can reduce renal plasma flow and glomerular filtration rate through hyperviscosity (22). Pathologically elevated HCT (>65%), a commonly used international threshold, is considered a major driver of increased blood viscosity (23). In our cohort, although HCT as a continuous variable was significantly higher in the AKI group (*p* < 0.001), the categorical analysis of markedly elevated HCT (> 65%) did not reach statistical significance (*p* = 0.375). This lack of association may be partly attributed to the limited statistical power, as only 35 patients met this extreme threshold. However, a more nuanced interpretation suggests that in the context of acute neurocritical illness, which is characterized by systemic inflammation and hemodynamic instability, the kidney may become hypersensitive to even moderate increases in blood viscosity. Consequently, the pathological impact of erythrocytosis might manifest at lower thresholds than those typically defined for chronic high-altitude polycythemia. However, this interpretation remains speculative, and prospective studies incorporating direct viscosity measurements and renal blood flow data are required for definitive validation.

We evaluated several alternative explanations for the link between elevated hemoglobin and AKI. Although systemic stress and disease severity could theoretically influence erythropoietin levels, our multivariate analysis (Table 3) controlled for these factors using APACHE II and GCS scores. While the independent association between hemoglobin and AKI remained robust after this adjustment, residual confounding related to unmeasured aspects of disease severity or its dynamic progression cannot be entirely excluded. Furthermore, hemoconcentration from dehydration is an unlikely primary confounder, as no significant differences between the AKI and non-AKI groups in serum sodium or the BUN/creatinine ratio (Table 1). Finally, the possibility that residual renal impairment hindered erythropoietin clearance was minimized by excluding patients with advanced CKD and confirming that baseline eGFR was comparable between the two groups. These findings suggest that the observed erythrocytosis likely reflects a primary physiological response to high-altitude hypoxia rather than secondary clinical interference.

Other identified risk factors for AKI, including older age, higher BMI, lower GCS score on admission, and hypotension, are consistent with findings from studies conducted in non-high-altitude regions (3, 24–26). Advanced age and higher BMI are known to impair renal reserve and increase susceptibility to ischemic or toxic injury. Low GCS scores may reflect more severe neurological and systemic stress, often accompanied by hemodynamic instability and higher exposure to nephrotoxic therapies. While multivariable regression linked contrast exposure to AKI, this association lost statistical significance in our PSM sensitivity analysis. It suggests that in our cohort, contrast exposure primarily serves as a surrogate marker for disease severity and intensive medical intervention, rather than acting as a strong and direct nephrotoxin. This finding aligns with the evolving clinical paradigm that differentiates contrast-associated AKI from true contrast-induced AKI (27). Conversely, the association between high-dose loop diuretics and AKI remained statistically significant in our PSM sensitivity analysis. This suggests that in our cohort, high-dose furosemide exposure is not merely a surrogate marker for disease severity, but rather an independent risk factor for renal injury. This finding underscores the clinical necessity of careful diuretic titration to avoid aggravating renal vulnerability in high-altitude neurocritical settings.

This study has several strengths. First, it is the first investigation to systematically evaluate AKI incidence and risk factors among neurocritical patients at high altitude, highlighting both shared and altitude-specific determinants such as erythrocytosis. Second, the study was conducted at the largest and most representative tertiary medical center in Xizang, ensuring the clinical relevance and generalizability of the findings to high-altitude populations.

However, several limitations should be noted. First, as a retrospective study, potential confounding factors and incomplete data cannot be completely avoided. Specifically, for patients whose baseline SCr was defined by the first hospital admission value, it is possible that some had already experienced a degree of renal impairment before the first measurement. This could lead to an overestimation of the baseline SCr and a subsequent underdiagnosis of AKI cases. Second, this was a single-center study, and multicenter validation is needed to confirm the generalizability of the results.

## 5 Conclusion

This study is the first to investigate acute kidney injury in neurocritical patients living at high altitude. AKI was common and associated with prolonged ventilation, longer ICU stay, higher mortality, and increased hospitalization costs. In addition to common predictors, both low and high hemoglobin levels were significantly associated with increased AKI risk. These findings suggest that erythrocytosis induced by high altitude may contribute to renal injury. Further prospective studies and experimental data are needed to confirm these results and guide AKI prevention and management in high-altitude neurocritical settings.

## Data Availability

The datasets presented in this article are not readily available because access requires clarification of the intended use and approval from the corresponding author. Requests to access the datasets should be directed to Nan Li, iculinan@163.com.
