# Supplementary material for: Incidence and risk factors of acute kidney injury among neurocritical patients in high-altitude regions: a retrospective cohort study
Source: Front Med (Lausanne). 2026 Apr 22;13:1756455. doi: 10.3389/fmed.2026.1756455 (PMC13144066; doi:10.3389/fmed.2026.1756455)
Supplement: Supplementary file 1 [file Supplementary_file_1.docx]

**Table S1. Detailed characterization of nephrotoxic exposures within 72 hours of ICU admission**

| Cumulative doses* | All  (n=390) | Non-AKI  (n=307) | AKI  (n=83) | p |
| --- | --- | --- | --- | --- |
| Mannitol |  |  |  |  |
| Usage (%) | 365 (93.6%) | 287 (93.5%) | 78 (94.0%) | 0.871 |
| Mannitol (g) | 350 (275, 400) | 350 (275, 400) | 338 (300, 375) | 0.927 |
| Loop diuretics |  |  |  |  |
| Usage (%) | 337 (86.4%) | 260 (84.7%) | 77 (92.8%) | 0.057 |
| Furosemide (mg) | 25 (10, 45) | 20 (10, 40) | 40 (20, 70) | <0.001 |
| NSAIDs |  |  |  |  |
| Usage (%) | 78 (20.0%) | 59 (19.2%) | 19 (22.9%) | 0.458 |
| Loxoprofen (mg) | 60 (60, 60) | 60 (60, 60) | 60 (60, 60) | 1.000 |
| Indomethacin (mg) | 100 (100, 100) | 100 (100, 100) | 100 (100, 100) | 1.000 |
| Vancomycin |  |  |  |  |
| Usage (%) | 44 (11.3%) | 31 (10.1%) | 13 (15.7%) | 0.155 |
| Vancomycin (g) | 1 (1, 1) | 1 (1, 1) | 1 (1, 1) | 1.000 |

AKI, acute kidney injury; NSAIDs, nonsteroidal anti-inflammatory drugs.

* Cumulative doses are calculated only among patients who received the specific medication.

**Table S2. Covariates of patients before and after propensity score matching for contrast exposure**

| Covariates | Unmatched cohort (n=390) | | | Matched cohort (1:2, n=232)* | | | |
| --- | --- | --- | --- | --- | --- | --- | --- |
|  | Contrast (n=85) | Control (n=305) | SMD | | Contrast (n=82) | Control (n=150) | SMD |
| Age (y) | 48 (12) | 52 (15) | 0.28 | | 49 (12) | 49 (14) | 0.07 |
| Male | 48 (56.5%) | 195 (63.9%) | 0.15 | | 46 (56.1%) | 85 (56.7%) | 0.01 |
| BMI (kg/m^2^) | 24.2 (3.19) | 23.9 (3.22) | 0.12 | | 24.3 (3.11) | 24.2 (3.76) | 0.01 |
| Hypertension | 40 (47.1%) | 129 (42.3%) | 0.10 | | 39 (47.6%) | 73 (48.7%) | 0.02 |
| Cerebrovascular injury ^a^ | 78 (91.8%) | 187 (61.3%) | 0.77 | | 75 (91.5%) | 139 (92.7%) | 0.04 |
| GCS score on admission | 9 (6, 14) | 9 (5, 15) | 0.01 | | 9 (5, 14) | 9 (5, 15) | 0.02 |
| APACHE Ⅱ score | 16.3 (5.5) | 16.2 (5.6) | 0.03 | | 16.2 (5.6) | 15.8 (5.2) | 0.07 |
| Baseline eGFR (mL/min·1.73m²) | 110 (102, 125) | 105 (93, 118) | 0.36 | | 109 (101, 123) | 108 (99, 122) | 0.08 |
| Hemoglobin (g/L) |  |  | 0.13 | |  |  | 0.04 |
| <100 | 2 (2.4%) | 12 (3.9%) |  | | 2 (2.4%) | 5 (3.3%) |  |
| 100-180 | 56 (65.9%) | 211 (69.2%) |  | | 54 (65.9%) | 100 (66.7%) |  |
| >180 | 27 (31.8%) | 82 (26.9%) |  | | 26 (31.7%) | 46 (30.7%) |  |

BMI, body mass index; GCS, Glasgow Coma Scale; APACHE II, Acute Physiology and Chronic Health Evaluation II; eGFR, estimated glomerular filtration rate; SMD, standardized mean difference.

^a^ including hemorrhagic stroke, ischemic stroke, hypertensive intracerebral hemorrhage, and aneurysmal subarachnoid hemorrhage.

* Due to the implementation of a 0.2 caliper width, some treated subjects were matched with fewer than two controls (variable-ratio matching) to maintain optimal covariate balance. Descriptive statistics for the matched control group were weighted accordingly.

**Table S3. Covariates of patients before and after propensity score matching for high-dose loop diuretic exposure**

| Covariates | Unmatched cohort (n=390) | | | Matched cohort (1:1, n=304) | | | |
| --- | --- | --- | --- | --- | --- | --- | --- |
|  | High-dose (n=167) | Low-dose/None (n=223) | SMD | | High-dose (n=152) | Low-dose/None (n=152) | SMD |
| Age (y) | 52 (15) | 50 (14) | 0.08 | | 51 (15) | 51 (14) | 0 |
| Male | 107 (64%) | 136 (61%) | 0.06 | | 97 (64%) | 101 (66%) | 0.06 |
| BMI (kg/m^2^) | 24.08 (3.10) | 23.86 (3.30) | 0.07 | | 24.08 (3.13) | 24.01 (3.40) | 0.02 |
| Hypertension | 76 (46%) | 93 (42%) | 0.08 | | 68 (45%) | 65 (43%) | 0.04 |
| Cerebrovascular injury ^a^ | 116 (69%) | 149 (67%) | 0.06 | | 106 (70%) | 104 (68%) | 0.03 |
| GCS score on admission | 8 (5, 12) | 13 (6, 15) | 0.62 | | 8.6 (4.0) | 9.0 (4.4) | 0.09 |
| APACHE Ⅱ score | 17.2 (5.4) | 15.4 (5.6) | 0.32 | | 16.8 (5.2) | 16.5 (5.4) | 0.06 |
| Baseline eGFR (mL/min·1.73m²) | 104 (93, 120) | 107 (95, 120) | 0.02 | | 107 (20) | 107 (19) | 0.03 |
| Hypotension | 25 (15%) | 31 (14%) | 0.03 | | 24 (16%) | 18 (12%) | 0.09 |
| Hemoglobin (g/L) |  |  | 0.27 | |  |  | 0.05 |
| <100 | 5 (3.0%) | 9 (4.0%) |  | | 5 (3.3%) | 4 (2.6%) |  |
| 100-180 | 104 (62%) | 163 (73%) |  | | 100 (66%) | 103 (68%) |  |
| >180 | 58 (35%) | 51 (23%) |  | | 47 (31%) | 45 (30%) |  |

BMI, body mass index; GCS, Glasgow Coma Scale; APACHE II, Acute Physiology and Chronic Health Evaluation II; eGFR, estimated glomerular filtration rate; SMD, standardized mean difference.

^a^ including hemorrhagic stroke, ischemic stroke, hypertensive intracerebral hemorrhage, and aneurysmal subarachnoid hemorrhage.
